# Effectiveness of an intervention to facilitate the implementation of healthy eating and physical activity policies and practices in childcare services: a randomised controlled trial

**DOI:** 10.1186/s13012-015-0340-z

**Published:** 2015-10-25

**Authors:** Jannah Jones, Rebecca Wyse, Meghan Finch, Christophe Lecathelinais, John Wiggers, Josephine Marshall, Maryann Falkiner, Nicole Pond, Sze Lin Yoong, Jenna Hollis, Alison Fielding, Pennie Dodds, Tara Clinton-McHarg, Megan Freund, Patrick McElduff, Karen Gillham, Luke Wolfenden

**Affiliations:** 1 Hunter New England Population Health, Locked Bag 10, Wallsend, NSW 2287 Australia; 2 School of Medicine and Public Health, University of Newcastle, Callaghan, NSW 2308 Australia; 3 Hunter Medical Research Institute, Newcastle, NSW 2300 Australia; 4 Priority Research Centre for Health Behaviour, University of Newcastle, Callaghan, NSW 2308 Australia

**Keywords:** Obesity prevention, Healthy eating, Physical activity, Childcare, Implementation

## Abstract

**Background:**

The primary aim of this study was to evaluate the effectiveness of an intervention to increase the implementation of healthy eating and physical activity policies and practices by centre-based childcare services. The study also sought to determine if the intervention was effective in improving child dietary intake and increasing child physical activity levels while attending childcare.

**Methods:**

A parallel group, randomised controlled trial was conducted in a sample of 128 childcare services. Intervention strategies included provision of implementation support staff, securing executive support, staff training, consensus processes, academic detailing visits, tools and resources, performance monitoring and feedback and a communications strategy. The primary outcome of the trial was the proportion of services implementing all seven healthy eating and physical activity policies and practices targeted by the intervention. Outcome data were collected via telephone surveys with nominated supervisors and room leaders at baseline and immediately post-intervention. Secondary trial outcomes included the differences between groups in the number of serves consumed by children for each food group within the Australian Guide to Healthy Eating and in the proportion of children engaged in sedentary, walking or very active physical activity assessed via observation in a random subsample of 36 services at follow-up.

**Results:**

There was no significant difference between groups for the primary trial outcome (*p* = 0.44). Relative to the control group, a significantly larger proportion of intervention group services reported having a written nutrition and physical activity policy (*p* = 0.05) and providing adult-guided activities to develop fundamental movement skills (*p* = 0.01). There were no significant differences between groups at follow-up on measures of child dietary intake or physical activity.

**Conclusions:**

The findings of the trial were equivocal. While there was no significant difference between groups for the primary trial outcome, the intervention did significantly increase the proportion of intervention group services implementing two of the seven healthy eating and physical activity policies and practices. High levels of implementation of a number of policies and practices at baseline, significant obesity prevention activity in the study region and higher than previously reported intra-class correlation of child behaviours may, in part, explain the trial findings.

**Trial registration:**

Australian Clinical Trials Registry (reference ACTRN12612000927820).

## Background

Overweight and obesity in childhood increases the likelihood of adult obesity and its comorbidities, including cardiovascular disease, type-2 diabetes and certain cancers [1, 2]. Inadequate physical activity and poor nutrition are key risk factors for overweight and obesity, with such risk behaviours beginning to develop in the early years of a child’s life, prior to commencing formal schooling [3]. As such, population-level interventions that aim to increase physical activity and improve diet quality during early childhood have been recommended [3–8].

Childcare services are an important setting for the delivery of obesity prevention interventions, given their potential to support population-level improvements in child diet and physical activity [9]. In countries such as Australia, the United States and the United Kingdom, over half of all children aged 0 to 5 years spend a large proportion of their waking hours each day in centre-based childcare services [9–12]. Best practice guidelines [13, 14] as well as standards for licensing and accreditation [15] recommend that childcare services implement policies and practices known to improve the quality of children’s diet and increase the time children spend being physically active while in care. Specifically, such guidelines recommend that services develop centre-based nutrition and physical activity policies, implement guidelines for foods brought from home or provided by the service, provide structured fundamental movement skill activities, ensure staff role model healthy eating and physical activity behaviours, limit the provision of sweetened drinks and limit opportunities for screen time [13, 14, 16, 17]. Such recommendations are supported by empirical research which suggests that implementation of such policies and practices improve child diet and physical activity while in care and can prevent excessive weight gain [18–20].

Despite evidence to support the effectiveness of such guidelines, research suggests that their implementation by childcare services is substandard. Studies from the United States found that less than 60 % of childcare services had a written physical activity policy [14] with some services providing food which contributed less than 17 % of children’s recommended dietary intake for vegetables [21]. Similarly, Australian research suggests that less than 50 % of childcare services have a written physical activity policy [22], only 46–60 % of services program time each day for fundamental movement skill development [22] and less than 5 % of childcare services provide adequate serves of vegetables, as recommended by national dietary guidelines [23]. Further, 25 % of Australian childcare services provide daily opportunities for sedentary screen time [22], 48 % provide sweetened drinks [24] and 60 % allow children to bring lunchboxes containing more than one serve of high fat, salt or sugary foods or drinks [25].

The authors are aware of three randomised controlled trials that have been conducted with the aim of increasing the implementation of healthy eating and physical activity policies and practices in childcare services. The trials were conducted from 2008 to 2014 in the United States and Australia and involved between 17 and 82 childcare services [26–28]. Two of the trials sought to implement the United States Nutrition and Physical Activity Self-Assessment for Child Care program using a variety of implementation strategies including environmental self-assessment, selection of areas for change, educational workshops for childcare service staff and parents, targeted technical assistance, consultation visits and printed resources [26, 28]. Evaluated using an environmental observation score, both failed to significantly increase service implementation of nutrition and physical activity practices [26, 28], but one significantly improved the quantity and quality of service nutrition and physical activity policies [28]. The third trial delivered an intervention comprising of professional development for childcare staff, resources and access to health promotion staff [27]. Hardy and colleagues significantly increased the frequency of fundamental movement skill sessions, yet there were no between-group differences on five other measures of the physical activity environment nor was the intervention successful in achieving change to service food policies or in-service food activities [27].

While several frameworks exist to guide the development and implementation of interventions to more effectively increase the implementation of policies and practices in childcare (e.g. [29, 30]), few randomised trials have been conducted to assess the effectiveness of implementation strategies in this setting. Implementation research conducted in clinical settings has found that trials utilising multiple intervention strategies and guided by theory are more likely to be effective in changing practice [31]. More extended periods of intervention support have been recommended to increase effectiveness [27]. However, previous trials conducted in the childcare setting have either utilised a limited number of such strategies [27] trialled interventions without the guidance of theoretical frameworks [27, 28] or were delivered over a short period of time (less than 7 months) [26–28]. Further two of the three studies delivered interventions to only a small number of childcare services (15 services or less), limiting their capacity to guide approaches aimed at achieving population-level service improvements [27, 28].

The primary aim of the trial was to assess whether a multicomponent intervention, delivered over 12 months was effective in increasing the proportion of centre-based childcare services implementing healthy eating and physical activity policies and practices. The study also sought to determine if the intervention was effective in improving child dietary intake and increasing child physical activity levels while attending childcare and, as a potential adverse effect, if it increased the occurrence of injury among staff or children.

## Methods

The trial was funded by the Australian National Preventive Health Agency (reference 95WOL2011) and was prospectively registered with the Australian New Zealand Clinical Trials Registry (reference ACTRN12612000927820). Ethical approval to conduct the study was obtained from the Hunter New England (reference 12/08/15/5.01) and the University of Newcastle (reference H-2012-0321) human research ethics committees. The research is reported in accordance with the requirements of CONSORT Statement [32].

### Design and setting

A detailed protocol for the trial has been published elsewhere [33]. A parallel group randomised controlled trial was conducted in 128 centre-based childcare services in the Hunter region of New South Wales, Australia, from August 2012 to July 2014.

### Participants

Centre-based childcare services included pre-schools and long day-care services. Services in the region were ineligible if they: catered exclusively for children requiring specialist care (less than 1 % of services), provided all on-site meals to children (approximately 30 % of services) or were fully government funded (approximately 3 % of services), as the ethical clearance and intervention design were not appropriate for such services.

### Recruitment procedures

Nominated supervisors (service managers) at all eligible services were contacted by a research assistant and invited to provide consent: (1) for their service to participate in the study, (2) for their own participation in a computer-assisted telephone interview (CATI) survey and (3) for one of their room leaders (head teacher caring for 3 to 5 year-old children) to be contacted and invited to participate in a CATI survey. A subsample of nominated supervisors were randomly selected by a research assistant using a random number function and invited to provide consent for their service to participate in a 1-day, post-intervention observation to assess child dietary intake and physical activity behaviours.

### Randomisation and allocation

Following the completion of baseline data collection, childcare services were randomly allocated to either the intervention or control condition by a research assistant using a random number function in a 1:1 (intervention: control) ratio. Services were not blind to study allocation.

### Intervention group

Briefly, the 12 month intervention aimed to increase childcare service implementation of healthy eating and physical activity policies and practices. The policies and practices were developed based on best practice Australian healthy eating and physical activity guidelines for the childcare setting [13] and those shown to be associated with child healthy eating and physical activity behaviours [16, 17]. The healthy eating and physical activity policies and practices implemented by services included the following:

1. Development of written nutrition and physical activity policies
2. Staff monitoring of children's lunchboxes every day against written nutritional guidelines and provision of feedback to parents when a non-compliant food was packed
3. Provision of water or reduced fat milk (for children over the age of 2 years) only
4. Staff role modelling of physically active play and healthy eating every day
5. Staff provision of prompts and positive comments to children to encourage physical activity and healthy eating every day
6. Provision of adult-guided fundamental movement skill development activities every day for at least 75 % of children
7. Restriction of sedentary screen time to less than weekly.

The design of the intervention to support implementation of the policies and practices utilised Damschroder’s Consolidated Framework for Implementation Research [34]. The Framework integrates 19 theoretical models and is composed of five major domains identified as influential in successful intervention implementation. The application of the relevant constructs to the intervention has been published as part of the study protocol [33]. The intervention consisted of eight evidence-based strategies to facilitate childcare service implementation of the healthy eating and physical activity policies and practices [35–39]. The intervention strategies included the following:

1. Implementation support staff [40]—The research team provided each service with a support staff member who provided on-going implementation support and positive reinforcement via face-to-face visits, telephone and email contact. Implementation support staff members had tertiary qualifications in nutrition and dietetics, health education and psychology and had previous experience in delivering similar initiatives in the childcare setting.
2. Securing executive support [41, 42] – Nominated supervisors were asked to lead the development and implementation of nutrition and physical activity policies, co-facilitate training workshops with implementation support staff and communicate expectations regarding the implementation of policies and practices to childcare service staff during staff meetings.
3. Provision of staff training [43]—A series of three 1-h training workshops which focused on policy and practice implementation were provided on-site to childcare service staff and included both didactic and interactive components.
4. Employment of consensus processes [35, 44]— Following each staff training workshop, implementation support staff facilitated a discussion with nominated supervisors and childcare service staff to reach group agreement regarding an implementation strategy for the targeted policies and practices.
5. Provision of academic detailing visits [45, 46]—Following each staff training workshop, an academic detailing visit was conducted which involved support staff observing and providing immediate feedback to childcare service staff as they implemented the practices targeted by the intervention.
6. Provision of tools and resources [40] – All services received an electronic and hardcopy package of tools and resources to support childcare service staff to implement the healthy eating and physical activity policies and practices.
7. Performance monitoring and feedback [47, 48]—Verbal and written feedback describing service progress toward implementation of the targeted policies and practices was delivered at six intervals throughout the 12 month intervention, with feedback based on information collected via the baseline CATI, telephone contacts and face-to-face visits.
8. Employment of a communications strategy [49]—Services received hard copy and electronic bimonthly newsletters which communicated key messages relating to the healthy eating and physical activity policies and practices. Services that implemented all policies and practices received a certificate of recognition, were acknowledged in newsletters and were used as case study examples.

### Control group

Participating services randomised to the control group received three newsletters at the commencement, mid-point and conclusion of the 12 month intervention. The newsletters were provided in hard copy and electronic formats and contained information on healthy eating and physical activity unrelated to the specific policies and practices targeted by the intervention. Control group services did not receive any other intervention from the research team during the study period.

### Data collection and measures

Surveys administered via CATI were conducted with the nominated supervisor and a room leader caring for children 3 to 5 years. Baseline data collection occurred between August and November 2012 and assessed childcare service characteristics and healthy eating and physical activity policies and practices. Follow-up CATI surveys were conducted immediately post-intervention between May and July 2014 and assessed healthy eating and physical activity policies and practices, staff and child injury, and in the intervention group, the acceptability of the intervention.

#### Service characteristics

Nominated supervisors were asked to report on the following: service days and hours of operation, type of service (pre-school or long day-care service), postcode, number of enrolled and attending children, number of primary contact teaching staff and whether any Aboriginal and/or Torres Strait Islander children were enrolled. The items used to assess service characteristics have been used in other Australian surveys of childcare services conducted by the research team [22, 24, 50].

#### Primary trial outcome

##### Healthy eating and physical activity policy and practice implementation

The primary trial outcome was the difference over time between groups in the proportion of services implementing all seven healthy eating and physical activity policies and practices. The primary trial outcome represents service achievement of “best practice”, maximising the potential of the service to support child healthy eating and physical activity.

Both nominated supervisors and room leaders were asked to report on their service’s implementation of the seven healthy eating and physical activity policies and practices using items validated in a previous sample of 42 Australian childcare services [51]. Nominated supervisors were asked to report on the implementation of whole-of-service policies and practices. Room leaders were asked to report on the implementation of specific healthy eating and physical activity policies and practices within their room. Each survey item and its respective percent agreement and Kappa value (*K*) are listed below in order to provide an indication of the level of agreement between nominated supervisor report and independent observation [51].

1. Presence of written nutrition (75 %, *K* = 0.50) and physical activity policies (79 %, *K* = 0.59)
2. Staff monitoring of children’s lunchboxes against written nutritional guidelines (84 %, *K* = 0.69) and provision of feedback to parents when a non-compliant food is packed (68 %, *K* = 0.34)
3. Provision of water (89 %, *K* = 0.78) or reduced fat milk only (79 %, *K* = 0.57) to children
4. Staff role modelling of physically active play (69 %, *K* = 0.39) and healthy eating (94 %, *K* = 0.89) every day
5. Staff provision of prompts and positive comments to children to encourage physical activity (80 %, *K* = 0.60) and healthy eating (86 %, *K* = 0.71) every day
6. Provision of adult-guided fundamental movement skill development activities (53 %, *K* = 0.06) every day to at least 75 % of children (60 %, *K* = 0.20)
7. Restriction of sedentary screen time (58 %, *K* = 0.17) to less than weekly.

#### Secondary trial outcomes

In order to assess if the hypothesised improvements in implementation of the healthy eating and physical activity policies and practices was sufficient to yield improvements to child diet and physical activity while attending childcare, observations of child dietary intake and physical activity levels were undertaken. The 1-day observation was conducted during core service hours (9 am–3 pm) in a random subsample of intervention and control group childcare services at follow-up. One of four trained observers attended each service to observe both child dietary intake and physical activity during the 1-day observation. Observers did not participate in the delivery of the intervention and were blind to service group allocation.

##### Child dietary intake

Secondary trial outcomes included the differences between groups at follow-up in the mean number of serves consumed by children for each food group within the Australian Guide to Healthy Eating (vegetables, fruit, grains, meat and meat alternatives, milk, yoghurt and cheese and discretionary foods). Child dietary intake was assessed during the 1-day observation using a modified version of the Dietary Observation for Child Care protocol [52]. The Dietary Observation for Child Care is a validated method for recording child-level dietary intake in 2 to 5 year-olds [52] and has been used extensively in the childcare setting [21, 53, 54]. Dietary intake was assessed in three children per service by an observer who visually estimated and recorded all types and portions of foods and drinks provided to and consumed by the children, along with amounts remaining after finishing a meal or snack [52]. This was recorded for every food or drink item supplied by parents in the child’s lunchbox and offered to the child during the observation period. The children were randomly selected by asking the room leader at each service to identify the three children with the most recent birthdays. Following the completion of the observation, the numbers of serves for each of the Australian Guide to Healthy Eating food groups was generated by a qualified dietitian. The number of serves consumed for each food group was calculated using the weight of the food according to a nutrient database [55] and the standard serve size of the food according to the Australian Guide to Healthy Eating [56]. Discretionary foods were classified using the Australian Guide to Healthy Eating with reference to the Australian Bureau of Statistics Discretionary Food List where unclear [57].

Observers were trained according to the Dietary Observation for Child Care protocol [52]. Prior to undertaking the observations, the observers completed a 20-food certification test. The observer results were compared to the actual measured amounts of foods and a tolerance level was set for each of the 20 items. The observers correctly described more than 90 % of items within the test and reached between 75 and 100 % agreement with actual measured amounts for the 20 food and drink items.

##### Child physical activity

Secondary trial outcomes included the differences between groups at follow-up in the proportion of children engaged in sedentary, walking or very active physical activity during all observations, structured physical activity and outdoor free play sessions. Child physical activity levels were assessed at the same 1-day observation by the same observer, using a modified version of the System for Observing Play and Leisure in Youth (SOPLAY) tool and protocol [58]. SOPLAY is a standardised instrument for assessing physical activity levels in recreational settings using systematic, momentary time sampling of a predetermined area [58]. SOPLAY has been found to be both valid and reliable in school-aged children [59] and has been previously used to assess physical activity in the childcare setting [60]. The observer coded all structured physical activity and outdoor free play sessions that occurred between 9 am and 3 pm at each service. Prior to the commencement of each physical activity session, observers recorded key aspects of the physical environment including location (inside or outside), type of session (structured physical activity or free play), scan start time and any equipment available for use. During each scan, the observers assessed the level of child physical activity by counting the number of children engaged in sedentary, walking or very active physical activity in 10-min intervals for the duration of each session.

Observers were trained according to the standardised SOPLAY protocol [58]. The SOPLAY assessment DVD was used to assess each observer’s ability to independently scan and code physical activity levels quickly and accurately. Of the 28 video clips in the assessment, observers must have correctly counted the number of people engaged in either sedentary, walking or very active activity in each clip to receive one point. Scores ranged between 61 and 71 %.

#### Other measures

##### Adverse effects—staff and child injury

Given an increase in child physical activity levels could potentially increase the risk of child injury [61], nominated supervisors in both the intervention and control groups were asked to report on the number of staff and children involved in adverse events in their service. Adverse events were defined as injuries requiring documentation during the previous 12 months.

##### Acceptability of the intervention

Nominated supervisors and room leaders in the intervention group were asked to respond on a four-point Likert scale (strongly agree, agree, disagree, strongly disagree) to a series of statements assessing the acceptability of the intervention resources, training and support provided to services.

##### Delivery of the intervention

The delivery of each of the eight intervention strategies was assessed by an independent research assistant using project records maintained by each implementation support staff member.

##### Blinding of CATI interviewers

CATI interviewers did not participate in the delivery of the intervention and were blind to service group allocation. To assess whether blinding was maintained, after collection of follow-up data, interviewers were asked to nominate the group to which they thought the service had been allocated.

##### Context

For descriptive purposes and to aid an assessment of any external influences on the trial findings, a systematic search was conducted to describe the context in which the trial was conducted [62, 63]. Local news archives, websites of national and New South Wales health and education departments, accreditation standards and national healthy eating and physical activity guidelines were reviewed to identify the existence of or changes in government policy, standards, funded programs, or guidelines that may influence the healthy eating and physical activity environments of childcare services. The search included the 12 months prior to and during the 12 month intervention.

### Sample size calculations

#### Primary trial outcome

Based on previous research, a 20 % study attrition rate of services was anticipated [50]. Given this, recruitment of 128 services into the trial at baseline would be sufficient to provide follow-up data from approximately 102 childcare services (51 per group) and enable the detection of an absolute difference between groups in the proportion of services implementing all policies and practices of 27 with 80 % power and an alpha value of 0.05. This was based on an expected prevalence of control group services implementing all policies and practices at follow-up of 25 %.

#### Secondary trial outcomes

Assuming a consent rate of 80 %, inviting a random subsample of 42 services to participate in the post-intervention observations would be sufficient to provide data from approximately 34 childcare services (17 services per group). This would enable the detection of an absolute difference between groups in very active physical activity of 4.3 % with 80 % power, an alpha of 0.05 and based on an intra-class correlation coefficient (ICC) of 0.02. This was based on estimations of four physical activity sessions per service, four 10-min scans per session and 20 children per 10-min scan. This sample was also sufficient to detect an absolute difference between groups in the mean number of serves for each food group of 0.3 serves with 80 % power, an alpha of 0.05 and based on an ICC of 0.02. This was based on estimations of three children being observed at each service (51 children per group) and a standard deviation of 0.5.

### Statistical analyses

All statistical analyses were performed using SAS (version 9.3) statistical software. All statistical tests were two tailed with an alpha value of 0.05.

#### Service characteristics

Descriptive statistics were used to describe the service characteristics of intervention and control group services at baseline. Socioeconomic characteristics were determined using service postcodes, which were classified as being in the top or bottom 50 % of New South Wales according to the Socio-economic Indices for Areas (SEIFA). Geographic characteristics of the service locality were classified as either urban or rural according to the Australian Statistical Geography Standard.

#### Healthy eating and physical activity policy and practice implementation

The primary trial outcome was analysed under an intention-to-treat framework using all available data. A logistic regression model was developed to determine group-by-time changes in the proportion of services implementing all healthy eating and physical activity policies and practices from baseline to follow-up. The logistic regression model included terms for time, group (intervention or control) and group-by-time interaction. A sensitivity analysis was performed by imputing baseline observations at follow-up for missing data. The same method of analysis (using six separate logistic regression models) was used to assess group-by-time changes in the following subgroups: service type (pre-school or long day-care service), socioeconomic characteristics (top or bottom 50 % of New South Wales) and geographic characteristics (urban or rural). As the study was not powered to test any hypotheses relating to such subgroups, these results are provided for descriptive purposes only. The following post hoc exploratory analyses were also performed: first, separate logistic regression models were used to determine group-by-time changes in the proportion of services implementing each of the individual policies and practices from baseline to follow-up. Second, a linear regression model was used to assess whether there was a significant difference over time between groups in the mean number of policies and practices implemented.

#### Child dietary intake

The amount of food consumed by each child was calculated using the food consumption equation, defined as: amount served less (amount remaining ± amount wasted or added) [52]. Descriptive statistics were used to assess child dietary intake data according to each of the Australian Guide to Healthy Eating food groups. A linear regression model was used to assess whether there was a significant difference between groups at follow-up in the mean number of serves for each food group (vegetables; fruit; grains; meat and meat alternatives; milk, yoghurt and cheese and discretionary foods). The model was adjusted for potential clustering effect.

#### Child physical activity

Descriptive statistics were used to assess the proportion of observations of the children’s physical activity levels. A logistic regression model was developed to assess whether there was a significant difference between groups at follow-up in the proportion of children engaged in sedentary, walking or very active physical activity. A generalised estimating equation (GEE) framework was utilised to account for potential clustering effects of the service (level one) and the SOPLAY session (level two). Analyses were performed on all observations, as well as on subgroups of the data including the type of physical activity (structured physical activity or outdoor free play session).

#### Acceptability of the intervention

Descriptive statistics were used to assess the delivery and acceptability of the intervention. Acceptability data was calculated using the percentage of nominated supervisors and room leaders that reported either “strongly agree” or “agree” to each item.

## Results

### Service characteristics

**Fig. 1:**
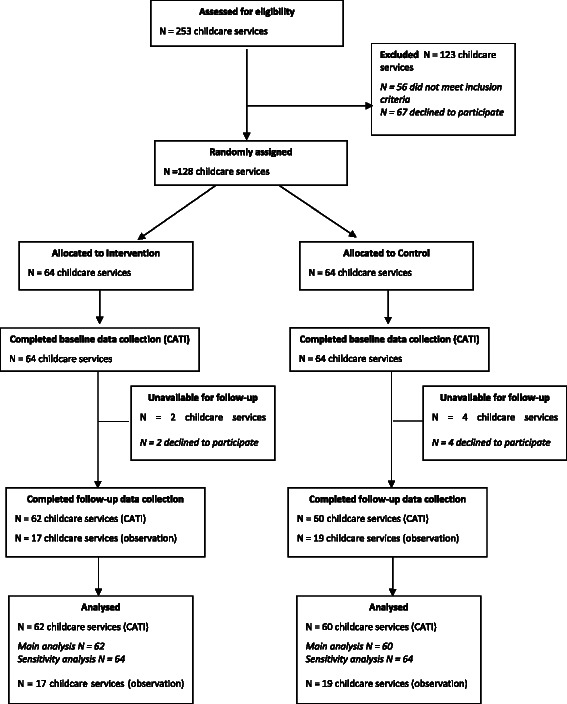
Participant recruitment and retention by group

Of the 253 childcare services in the study region, 128 (65 %) nominated supervisors consented for their service to participate in the study. Of these, 120 services (95 %) provided follow-up data (Fig. 1). The baseline characteristics of intervention and control group services that completed the CATI survey at both baseline and follow-up are described in Table 1. There were no differences between the characteristics of services that provided follow-up data and those that did not (*p* = 0.22–1.00). A randomly selected subsample of 42 nominated supervisors were invited to participate in a 1-day observation at follow-up, with 36 (86 %) consenting. There were no differences between groups in the baseline characteristics of services that did and did not consent to participate in the observations.

**Table 1:** Baseline characteristics of services included in the main outcome analyses by group

Characteristic		Intervention group *n* = 62	Control group *n* = 60
		% (95 % CI)	% (95 % CI)
Service operates 5 days per week	Yes	90 (83, 98)	98 (95, 100)
Type of service^a^	Pre-school	52 (39, 64)	53 (41, 66)
	Long day-care service	50 (37, 63)	50 (37, 63)
Children of aboriginal and/or Torres Strait Islander background enrolled	Yes	68 (56, 80)	78 (67, 89)
Service socio-economic area	Top 50 % of New South Wales	30 (18, 42)	27 (16, 39)
	Bottom 50 % of New South Wales	70 (58, 82)	73 (61, 84)
Service geographical location	Urban	50 (37, 63)	59 (46, 72)
	Rural	50 (37, 63)	41 (28, 53)
		Mean (SD)	Mean (SD)
Hours of operation		8.7 (2.0)	8.7 (1.7)
Number of children enrolled		77.6 (37.4)	86.7 (41.5)
Number of primary contact teaching staff		7.3 (4.1)	8.8 (4.6)

^a^5% of services identified as both a pre-school and long day-care service

### Primary trial outcome

#### Healthy eating and physical activity policy and practice implementation

There was no significant difference over time between groups in the proportion of services implementing all healthy eating and physical activity policies and practices, the primary trial outcome (*p* = 0.44) (Table 2). Relative to the control group, a significantly larger proportion of intervention group services reported having a written nutrition and physical activity policy (*p* = 0.05) and providing adult-guided fundamental movement skill development activities (*p* = 0.01) (Table 3). There was a significant difference between groups in the mean number of healthy eating and physical activity policies and practices implemented over time, favouring the intervention group (*p* = 0.05). Data from the subsample of services attended by observers at follow-up found significant improvement in the service implementation of staff role modelling of physically active play and healthy eating (*p* = 0.05) but not for other policies or practices (*p* = 0.27–0.96). There were no significant differences between the characteristics of services that were implementing all healthy eating and physical activity policies and practices at baseline and those that were not (*p* = 0.09–0.87).

**Table 2:** Change in proportion of services implementing all healthy eating and physical activity policies and practices over time by group—all services and by service type, socioeconomic characteristics and geographic characteristics

		Intervention group *n* = 62		Control group *n* = 60		Odds ratio (OR)	*p* value
		Baseline *n* (%)	Follow-up *n* (%)	Baseline *n* (%)	Follow-up *n* (%)	Intervention group versus control group (95 % CI)	
Main analysis	All services	15 (24)	27 (44)	12 (20)	22 (37)	1.33 (0.64, 2.76)	0.44
Sensitivity analysis	All services	15 (23)	27 (42)	13 (20)	23 (36)	1.29 (0.63, 2.64)	0.48
By service type	Pre-school	8 (25)	12 (38)	10 (31)	13 (41)	0.89 (0.33, 2.45)	0.83
	Long day-care service	7 (23)	15 (48)	3 (10)	11 (37)	1.67 (0.59, 4.73)	0.33
By socio-economic characteristics	Top 50 % of New South Wales	5 (28)	9 (50)	4 (25)	6 (38)	1.65 (0.42, 6.59)	0.48
	Bottom 50 % of New South Wales	10 (24)	16 (38)	8 (19)	16 (37)	1.05 (0.44, 2.54)	0.91
By geographic characteristics	Urban	8 (27)	16 (53)	9 (26)	15 (43)	1.52 (0.57, 4.07)	0.40
	Rural	7 (23)	9 (30)	3 (13)	7 (29)	1.04 (0.32, 3.41)	0.95

**Table 3:** Changes in proportion of services implementing each of the healthy eating and physical activity policies and practices over time by group

Outcome	Intervention group *n* = 62		Control group *n* = 60		
	Baseline *n* (%)	Follow-up *n* (%)	Baseline *n* (%)	Follow-up *n* (%)	*p* value
1. Presence of written nutrition and physical activity policies	42 (68)	60 (97)	35 (58)	51 (85)	0.05
2. Staff monitoring of children’s lunchboxes against written nutritional guidelines and provision of feedback to parents when a non-compliant food is packed^a^	48 (81)	46 (78)	45 (79)	46 (81)	0.69
3. Provision of water or reduced fat milk only to children	52 (84)	57 (91)	54 (90)	53 (88)	0.32
4. Staff role modelling of physically active play and healthy eating every day	54 (87)	51 (82)	48 (80)	48 (80)	0.71
5. Staff provision of prompts and positive comments to children to encourage physical activity and healthy eating every day	58 (94)	54 (87)	56 (93)	52 (87)	0.95
6. Provision of adult-guided fundamental movement skill development activities every day to at least 75 % of children	43 (69)	50 (81)	44 (73)	35 (58)	0.01
7. Restriction of sedentary screen time to less than weekly	57 (92)	58 (94)	54 (90)	55 (92)	0.75

^a^Excludes six services (three intervention and three control) that began providing on-site meals to children following the commencement of the intervention

### Secondary trial outcomes

#### Child dietary intake

There were no significant differences between groups at follow-up in the mean number of serves consumed by children for each food group (*p* = 0.14–0.96) (Table 4).

**Table 4:** Mean number of serves consumed by children for each food group within the Australian Guide to Healthy Eating and proportion of children engaged in sedentary, walking or very active physical activity by group at follow-up

Food group		Intervention group	Control group	*p* value
		*n* = 41 children, mean (SD) serves	*n* = 49 children, mean (SD) serves	
Vegetables		0.1 (0.3)	0.2 (0.6)	0.32
Fruit		1.1 (1.1)	0.8 (0.7)	0.14
Grains (breads and cereals)		1.6 (0.5)	1.4 (0.8)	0.28
Meat and meat alternatives		0.1 (0.2)	0.1 (0.3)	0.67
Milk, yoghurt and cheese		0.7 (0.6)	0.7 (0.7)	0.96
Discretionary foods^a^		0.7 (0.6)	0.7 (0.7)	0.79
Physical activity level		*n* = 17 services, % (95 % CI) of observations	*n* = 19 services, % (95 % CI) of observations	
All observations	Sedentary	44.8 (41.5, 48.1)	49.2 (45.8, 52.5)	0.49
	Walking	29.1 (26.5, 31.7)	29.5 (27.2, 31.8)	
	Very active	26.1 (22.5, 29.8)	21.3 (17.7, 24.9)	
Structured physical activity	Sedentary	41.5 (31.1, 51.9)	41.4 (31.3, 51.4)	0.64
	Walking	18.2 (10.4, 26.1)	25.7 (19.0, 32.5)	
	Very active	40.3 (29.5, 51.0)	32.9 (23.1, 42.6)	
Outdoor free play	Sedentary	45.7 (42.4, 49.0)	51.1 (48.1, 54.2)	0.37
	Walking	32.1 (29.7, 34.5)	30.5 (27.9, 33.0)	
	Very active	22.2 (19.4, 25.1)	18.4 (15.3, 21.5)	

^a^Includes foods high in saturated fat and/or added sugars, added salt or low in fibre, for example, sweet biscuits, cakes, processed meats, confectionary, savoury pastries and potato chips

#### Child physical activity

There were no significant differences between groups at follow-up in the proportion of children engaged in sedentary, walking or very active physical activity during all observations (*p* = 0.54), structured physical activity (*p* = 0.64) and outdoor free play sessions (*p* = 0.37) (Table 4).

### Other measures

#### Adverse effects—staff and child injury

At follow-up in the intervention group, the mean number of staff injuries during the previous 12 months was 0.77 (confidence interval (CI) 0.49–1.06) and mean number of serious child injuries was 0.72 (CI 0.39–1.05). In the control group, the mean number of staff injuries during the previous 12 months was 0.84 (CI 0.42–1.26) and mean number of serious child injuries was 0.90 (CI 0.52–1.29). There was no significant difference between groups in the mean number of staff (*p* = 0.80) or child (*p* = 0.47) injuries during the previous 12 months.

#### Acceptability of the intervention

All nominated supervisors, and 98 % of room leaders, found the implementation support to be beneficial to their service (Table 5). Ninety-five percent of nominated supervisors and room leaders stated that ongoing implementation support would be useful, and just four nominated supervisors and three room leaders would have preferred less support throughout the 12-month intervention.

**Table 5:** Acceptability of the intervention strategies to nominated supervisors and room leaders included in the main outcome analyses

Measure (agree/strongly agree)	Nominated supervisor *n* = 62, *n* (%)	Room leader *n* = 62, *n* (%)
Found the implementation support to be beneficial to their service	62 (100)	61 (98)
Found the face-to-face support to be acceptable	62 (100)	60 (97)
Found the telephone support to be acceptable	61 (98)	54 (87)
Found the training regarding healthy eating and physical activity beneficial for staff	62 (100)	60 (97)
Found the discussions following each training session to reach consensus on changes to healthy eating and physical activity practices at our service to be acceptable	62 (100)	59 (95)
Found the academic detailing sessions helpful	62 (100)	59 (95)
Found the resources provided useful	62 (100)	60 (97)
Found the performance feedback acceptable	62 (100)	57 (92)
Found the bimonthly newsletters acceptable	62 (100)	59 (95)
Felt comfortable talking to staff about changes to service healthy eating and physical activity policies and practices	62 (100)	61 (98)
Ongoing implementation support would be useful	59 (95)	59 (95)
Would have liked more support over the past 12 months	5 (8)	9 (15)
Would have liked less support over the past 12 months	4 (6)	3 (5)

#### Delivery of the intervention

Table 6 shows the proportion of childcare services in the intervention group that received each of the intervention strategies. All services were offered and accepted 12 months of implementation support via telephone contact from an implementation support staff member. Implementation support was staggered based on staffing availability from January 2013 to April 2014. Ninety-four percent of nominated supervisors demonstrated executive support for the trial via co-facilitation of training workshops with implementation support staff and participation in consensus processes. Seventy-seven percent of services received all three staff training workshops and 76 % received all three academic detailing sessions. A total of 69 % of services received the full complement of all eight intervention strategies.

**Table 6:** Extent of delivery of intervention strategies to intervention group childcare services included in the main outcome analyses

Intervention strategy	*n* = 62, *n* (%)
Implementation support staff	
Service received offer of support by implementation support staff	62 (100)
Executive support	
Nominated supervisor demonstrated executive support (co-facilitated training workshops with implementation support staff and participated in consensus processes)	58 (94)
Consensus processes	
Discussion following each staff training workshop occurred	60 (97)
Staff training	
Training session 1 delivered	60 (97)
Training session 2 delivered	55 (89)
Training session 3 delivered	48 (77)
Academic detailing	
Visit 1 delivered	60 (97)
Visit 2 delivered	56 (90)
Visit 3 delivered	47 (76)
Tools and resources	
Service distributed with relevant resources	62 (100)
Performance monitoring and feedback	
Service received feedback at six intervals throughout intervention	61 (98)
Communications strategy	
Bimonthly newsletters distributed	62 (100)
Service received recognition via certificate or case study in newsletter	59 (95)
Received all intervention strategies	43 (69)

#### Blinding of CATI interviewers

At follow-up, interviewers correctly identified the services' group allocation in 70 % (*p* = <0.001) of nominated supervisor surveys and 57 % (*p* = 0.68) of room leader surveys.

#### Context

Throughout the 12 months prior to, and during the 12 month intervention, two government guidelines that may have influenced the healthy eating and physical activity environments of childcare services were introduced. First, the Australian Dietary Guidelines (including the Australian Guide to Healthy Eating) were revised and released in February 2013 [56]. Second, the National Accreditation Standards for the childcare sector (The National Quality Framework) came into effect in the 12 months prior to intervention delivery (January 2012) [15]. In addition to government guidelines, two government-sponsored funded programs were identified. The first, a state-based program known as “Munch & Move”, was implemented during the study period [64]. Project records show that 80 % of intervention group services and 12 % of control group services attended training in healthy eating and physical activity provided by the “Munch & Move” program during the study period. Secondly, Australia’s largest community-based obesity prevention program, known as “Good for Kids. Good for Life” was conducted in the study region from 2006 to 2011 [65]. Project records provided by the program show 45 % of intervention group services and 52 % of control group services attended training in healthy eating and physical activity provided by the “Good for Kids. Good for Life” program during the period from 2006 to 2011.

## Discussion

Internationally, this is one of a small number of randomised controlled trials conducted to test an intervention aimed at increasing healthy eating and physical activity policy and practice implementation in childcare services. The study did not find a significant intervention effect on the primary trial outcome, the proportion of intervention group services implementing all seven policies and practices. However, the intervention significantly increased the proportion of intervention group services implementing two of these policies and practices (written nutrition and physical activity policy and provision of adult-guided activities to develop fundamental movement skills) and the mean number of policies and practices implemented.

The study findings are similar to that of the previously conducted randomised controlled trials in the childcare setting. Ward and colleagues found a non-significant improvement of 11 % in service nutrition and physical activity environments, policies and practices; Hardy and colleagues demonstrated a significant improvement in the provision of one of the six physical activity practices targeted by the intervention (frequency of fundamental movement skills sessions per week increased from 1.3 to 3.2), and Alkon and colleagues found significant improvements in the quantity of service policies (mean policy score increased from 0.89 to 5.17 (nutrition) and 0 to 2.82 (physical activity)) but no improvement in any of the 14 nutrition and physical activity practices targeted by the intervention [26–28]. It was anticipated that the current trial would yield an improvement of at least 20 % in the primary trial outcome, given the substantial increase in the number of intervention components, the duration of intervention support relative to past interventions, and evidence of similar effects sizes for implementation interventions of similar intensities in other settings [66].

There are several factors that may have limited the effectiveness of the intervention on the primary trial outcome. First, the primary outcome was a composite measure, requiring implementation of all seven targeted policies and practices by services. However, five of the seven policies and practices were being implemented by 80 % or more of intervention group services at baseline, limiting scope for further improvements. Second, the trial did not exclude services who were already implementing all policies and practices at baseline (24 % of intervention services). For these services, the benefit of further intervention is likely to be minimal. Third, at follow-up, the proportion of control services implementing all policies and practices increased by 17 %. Such substantial improvement in implementation in control groups has not been reported by other trials [26–28]. Context evaluation suggests that co-intervention in the control group and other important contextual factors such as the introduction of national dietary guidelines and the National Accreditation Standards occurring at the time of the trial may have facilitated policy and practice implementation in the control group, reducing the likelihood of an intervention effect. Finally, some policies and practices were particularly difficult for some services to implement. Anecdotally, for example, implementation support staff reported that most services that were not providing feedback to parents when non-compliant foods were packed found this practice to be particularly challenging, citing concerns about adverse reactions from parents.

In the context of the limited impact of the intervention on implementation of policies and practices, it is perhaps unsurprising that the intervention did not yield significant improvements in child dietary intake or physical activity levels in care. However, while non-significant, the effect size achieved for child very active physical activity (>4 %) and fruit intake (>1/3 of a serve) was consistent with the effect size on which the study had been powered to detect a priori. However, the ICC found in this study was far higher (0.34 for physical activity and 0.11 for fruit) than that which was predicted for the study (0.02) on which sample size calculations were based. The high ICC substantially reduced the effective sample size of the current study. Future studies using similar observational procedures to assess child diet and physical activity may require larger samples in order to detect clinically meaningful effects.

Strengths of the study include the trial’s randomised design, delivery of the intervention to a large population of childcare services, high study retention and the inclusion of child behavioural measurements at follow-up. The inclusion of a comprehensive set of process measures also provided a rich source of information to interpret the study findings. However, a number of study limitations are worth noting. First, the study relied on the self-report of nominated supervisors and room leaders for the measurement of service policies and practices, which may have introduced biases such as social desirability bias. While the survey items have been validated in a sample of Australian childcare services, a number of practices had only slight, fair or moderate agreement (kappa <0.6) [49]. Future studies should look to conduct a more rigorous assessment of implementation, such as direct observation of service policies and practices. Second, the trial did not measure change in perceived barriers and enablers to implementing the policies and practices and, as such, any mechanisms that may have facilitated the outcome of the intervention could not be investigated. Third, the intervention was multi-component and the effectiveness of the individual intervention strategies was unable to be determined. Future studies could examine the effectiveness of individual intervention strategies. Fourth, process evaluation did not include an assessment of the timing of when childcare services implemented each policy or practice. As such, the exposure of individual children to each policy and practice is unknown, preventing any assessment of the impact of such exposure on child healthy eating and physical activity. Fifth, the study assessed child dietary intake and physical activity levels on one day at follow-up. Repeated dietary intake and physical activity observations conducted over multiple days may provide a more robust measure of these behaviours during attendance at childcare. Sixth, the study did not include services that provide on-site meals to children. Future research may consider evaluating the impact of an intervention targeting food-provision practices specific to such childcare services.

## Conclusions

Despite the limitations, the study represents an important contribution to the limited literature regarding the implementation of obesity prevention interventions in the childcare setting. The findings demonstrate that, among a group of services where policy and practice implementation was generally high at baseline, the investment of significant implementation support to achieve “best practice” implementation may not yield significant improvements in the proportion of services achieving this goal. Investment in implementation support, therefore, may be better directed at services where policies and practice implementation is poorer initially, and where they may be greater scope for improvement. Future research to test the effectiveness of the intervention on such services is warranted. Given the high acceptability of the intervention strategies, prospective interventions may consider utilising strategies that best address the reported barriers to policy and practice implementation. For example, the addition of intervention strategies to garner the support for healthy eating and physical activity policies and practices by parents and carers may improve the effectiveness of future interventions, given their influence on service operation and activities [67]. Finally, the intervention did improve some policies and practices, including the implementation of adult-guided activities to develop fundamental movement skills in children. An improvement in the implementation of fundamental movement skills activities was also noted in a randomised trial by Hardy and colleagues [27]. Common to both interventions was staff training, resources and the use of implementation support staff. Such findings suggest that these intervention strategies may be particularly effective in supporting the implementation of this practice and should be retained in future interventions.
